# Prognosis of locally advanced rectal cancer can be predicted more accurately using pre- and post-chemoradiotherapy neutrophil-lymphocyte ratios in patients who received preoperative chemoradiotherapy

**DOI:** 10.1371/journal.pone.0173955

**Published:** 2017-03-14

**Authors:** SooYoon Sung, Seok Hyun Son, Eun Young Park, Chul Seung Kay

**Affiliations:** 1 Department of Radiation Oncology, Seoul St. Mary's Hospital, College of Medicine, The Catholic University of Korea, Seoul, Republic of Korea; 2 Department of Radiation Oncology, Incheon St. Mary's Hospital, College of Medicine, The Catholic University of Korea, Seoul, Republic of Korea; Universita degli Studi di Napoli Federico II, ITALY

## Abstract

**Purpose:**

The neutrophil-lymphocyte ratio (NLR) has been suggested as an inflammation-related factor, but also as an indicator of systemic anti-tumor immunity. We aimed to evaluate the prognostic value of the NLR and to propose a proper cut-off value in patients with locally advanced rectal cancer who received preoperative chemoradiation (CRT) followed by curative total mesorectal excision (TME).

**Methods:**

A total of 110 rectal cancer patients with clinical T3-4 or node-positive disease were retrospectively analyzed. The NLR value before preoperative CRT (pre-CRT NLR) and the NLR value between preoperative CRT and surgery (post-CRT NLR) were obtained. Using a maximally selected log-rank test, cut-off values were determined as 1.75 for the pre-CRT NLR and 5.14 for the post-CRT NLR.

**Results:**

Patients were grouped as follows: group A, pre-CRT NLR ≤ 1.75 and post-CRT NLR ≤ 5.14 (n = 29); group B, pre-CRT NLR > 1.75 and post-CRT NLR ≤ 5.14, or pre-CRT NLR ≤ 1.75 and post-CRT NLR > 5.14 (n = 61); group C, pre-CRT NLR > 1.75 and post-CRT NLR > 5.14 (n = 20). The median follow-up time was 31.1 months. The 3-year disease-free survival (DFS) and overall survival (OS) rates showed significant differences between the NLR groups (3-year DFS rate: 92.7% vs. 73.0% vs. 47.3%, for group A, B, and C, respectively, p = 0.018; 3-year OS rate: 96.0% vs. 85.5% vs. 59.8%, p = 0.034). Multivariate analysis revealed that the NLR was an independent prognostic factor for DFS (p = 0.028).

**Conclusion:**

Both the pre-CRT NLR and the post-CRT NLR have a predictive value for the prognosis of patients with locally advanced rectal cancer treated with preoperative CRT followed by curative TME and adjuvant chemotherapy. A persistently elevated post-CRT NLR may be an indicator of an increased risk of distant metastasis.

## Introduction

Preoperative chemoradiation (CRT) followed by curative surgery including total mesorectal excision (TME), and adjuvant chemotherapy has been the standard treatment for patients with locally advanced rectal cancer for more than a decade [1–4]. Preoperative CRT was reported to induce downstaging in 50–60% of patients, and pathologic complete remission (pCR) in 10–20% of patients [5]. In contrast, about 40% of patients showed ypT3-4 or ypN+ disease after preoperative CRT [6,7]. The diverse range of responses to preoperative CRT leads to heterogenous prognosis in locally advanced rectal cancer. Predictive markers of the response to preoperative CRT are needed to predict the prognosis of patients with rectal cancer who received preoperative CRT.

The neutrophil-lymphocyte ratio (NLR) has been suggested as a prognostic factor in many solid tumors [8–11]. The NLR is an indicator of inflammatory status, representing both the neutrophil and lymphocyte counts. A persistent inflammatory state is known to induce tumorigenesis [12]. Interactions between tumor cells and inflammatory cells are crucial for tumor cell survival and proliferation [13]. Studies assessing the NLR reported that a high NLR was associated with a poor survival outcome. In patients with hepatocellular carcinoma (HCC), those with a high NLR showed worse disease-free survival (DFS) and overall survival (OS) [14,15]. In colon cancer, the preoperative NLR was reported to be an independent prognostic factor for OS [16].

Despite evidence in other solid tumors, the prognostic value of the NLR has not been elucidated in patients with rectal cancer who received preoperative CRT. Before the introduction of preoperative CRT, the NLR was suggested as a prognostic factor for survival in patients who were treated with only surgical resection [17]. Though a few studies also reported that the pre-treatment NLR may be a predictive marker in rectal cancer patients who received preoperative CRT, the cut-off values for the NLR were variable [18–20]. In addition, according to a recent meta-analysis, both the pre-treatment NLR and the post-treatment NLR might be considered prognostic factors [21]. In rectal cancer, the prognostic value of the NLR and proper cut-off values have not yet been fully investigated. To our knowledge, there has been no report about evaluation of both the pre-CRT and post-CRT NLRs for prognosis in patients with locally advanced rectal cancer who received preoperative CRT.

In this study, we aimed to assess the prognostic value of pre- and post-CRT NLRs, and to suggest the optimal cut-off values to predict DFS in patients with locally advanced rectal cancer who received preoperative CRT followed by curative TME and adjuvant chemotherapy.

## Materials and methods

### Patients

Eligibility criteria were as follows: histologically proven adenocarcinoma in the rectum or, in a few cases, radiologically confirmed rectal cancer; clinical T3/T4 or lymph node-positive disease based on evaluation before preoperative CRT; treatment with preoperative CRT followed by curative TME; no evidence of distant metastasis at the time of diagnosis; lymphocyte and neutrophil counts obtained before, during, and after preoperative CRT; and an Eastern Cooperative Oncology Group (ECOG) performance score of 0–2. Patients who had fever, signs of upper respiratory infection or urinary tract infection, or other underlying diseases, such as rheumatoid disease, coronary artery disease, or metabolic syndrome were excluded because these conditions could change the NLR level [22–24].

A total of 110 patients met the eligibility criteria for this study between February 2006 and December 2013. All patients underwent preoperative CRT and TME at Incheon St. Mary’s Hospital. The patients’ medical records were retrospectively reviewed after approval by the Institutional Review Board of Incheon St. Mary’s Hospital. The patients’ clinicopathological characteristics are described in Table 1.

**Table 1:** Clinicopathologic characteristics.

Characteristics	n (%)
Age*	59 (27~84)
Sex	
Men	74 (67.3%)
Women	36 (32.7%)
Pre-CRT NLR*	2.10 (0.53–10.63)
Post-CRT NLR*	3.23 (0.48–21.64)
Pre-CRT CEA, ng/mL *	3.84 (0.75–1018.20)
Tumor distance from anal verge	
≤ 5 cm	57 (51.8%)
> 5 cm	53 (48.2%)
Clinical T stage	
cT0-2	7 (6.4%)
cT3-4	103 (93.6%)
Clinical N stage	
cN0	7 (6.4%)
cN1-2	103 (93.6%)
Histology	
WD	17 (15.5%)
MD	84 (76.4%)
PD	9 (8.2%)
Downstage	
Yes	35 (31.8%)
No	75 (68.2%)
pCR	
Yes	11 (10.0%)
No	99 (90.0%)
Pathologic T stage	
ypT0-2	37 (33.6%)
ypT3-4	73 (66.4%)
Pathologic N stage	
ypN0	76 (69.1%)
ypN1-2	34 (30.9%)
Margin status	
Negative	100 (90.9%)
Positive	10 (9.1%)
LVI**	
Negative	82 (74.5%)
Positive	25 (22.7%)
PNI**	
Negative	83 (75.5%)
Positive	22 (20.0%)

* Median, range

**LVI and PNI were evaluated on 107 and 105 patients, respectively, because of missing values

Abbreviations: APR = abdominoperitoneal resection, CEA = carcinoembryonic antigen, CRT = chemoradiotherapy, LVI = lymphovascular invasion, MD = moderately differentiated, NLR = neutrophil-lymphocyte ratio, pCR = pathologic complete response, PD = poorly differentiated, PNI = perineural invasion, post-CRT NLR = NLR values after preoperative CRT, pre-CRT NLR = NLR values before preoperative CRT, SSR = sphincter-saving resection, WD = well differentiated

### Pre-treatment evaluation

Before preoperative CRT, all patients were assessed using staging workups. The patients' histories were taken, physical examination, complete blood count (CBC), blood chemistry, serum carcinoembryonic antigen (CEA) measurement, and colonoscopy were performed. Radiologic examination, including chest radiography, computed tomography (CT) scan of the abdomen and pelvis, chest CT scan, and pelvic magnetic resonance imaging (MRI) were performed. A portion of patients also underwent a positron emission tomography (PET) CT scan. Clinical stage was determined according to the seventh edition of the TNM classification of the American Joint Committee on Cancer (AJCC) [25].

### Preoperative CRT

All patients underwent three-dimensional radiotherapy (RT) with concurrent chemotherapy. RT was applied to the whole pelvis with 45 Gy in 25 fractions, with a boost to the primary tumor of 5.4 Gy in 3 fractions. In total 50.4 Gy in 28 fractions were irradiated over 5–6 weeks (median, 38 days; range, 35–45 days). Patients were treated with 3 portals, which were posterior and bilateral beams in the prone position to reduce the volume of irradiated bowel within the pelvis. The superior border was the lumbosacral junction and the lower border was the lowest margin of the obturator foramen or 3 cm distal to the tumor. The lateral border of the posterior beam was 1.5–2.0 cm lateral to the widest bony margin of the true pelvis. Fields of bilateral beams were bordered with the posterior margin of the symphysis pubis anteriorly, and 1 cm behind the posterior border of the sacrum posteriorly. The boost volume was 3 cm expansion from the primary tumor in the superior and inferior directions, and 2 cm expansion radially.

Chemotherapy regimens were either intravenous 5-fluorouracil (5-FU) or oral capecitabine. 5-FU was administered to 103 patients (93.6%) and capecitabine was administered to 7 patients (6.4%). 5-FU was administered at a dose of 425 mg/m^2^/day of 5-FU with 20 mg/m^2^/day of leucovorin during the first and fifth weeks of RT. Oral capecitabine was administered at a dose of 1,650 mg/m^2^/day daily during the whole period of RT.

### Surgery

All patients underwent curative TME and pelvic node sampling or dissection at 6–8 weeks after preoperative CRT. Surgery was performed by an experienced colorectal surgeon and surgical specimens were evaluated by experienced pathologists. Positive margin status was regarded as tumor cell involvement within 1 mm of the circumferential margin, or involvement directly on the resected distal or proximal margin. Histology, grade, margin status, lymphovascular invasion (LVI), perineural invasion (PNI), and pathologic stage were determined by pathologic examination.

### Adjuvant chemotherapy

All patients were considered for adjuvant chemotherapy regardless of pathologic stage. Ninety-nine patients (90.0%) were treated with 5-FU/leucovorin (LF). Six cycles of LF chemotherapy were infused for 5 days every 4 weeks. 5-FU/oxaliplatin/leucovorin (FOLFOX) chemotherapy was administered to 2 patients (1.8%) who were diagnosed with early recurrences after surgery and before initiation of adjuvant chemotherapy. Patients who were diagnosed with recurrences during adjuvant LF chemotherapy were treated with alternative chemotherapy regimens; these were FOLFOX or 5-FU/irinotecan/leucovorin (FOLFIRI). Tegafur/uracil and oral capecitabine were each administered to 1 patient (0.9%). Seven patients (6.4%) did not receive adjuvant chemotherapy due to the patient’s refusal or poor performance status.

### NLR

The NLR was calculated from the neutrophil and lymphocyte counts of the CBC, which was examined before preoperative CRT, weekly during CRT, and 4 weeks after CRT. The pre-CRT NLR was obtained using the CBC result before preoperative CRT, and the post-CRT NLR was obtained using the CBC result 4 weeks after CRT.

### Follow-up

Patients were followed up every 3 month for the first 2 years. Physical examinations, CBC, blood chemistry, and chest radiography were performed at every follow-up. CT scans for the abdomen, pelvis, and chest were performed every 6–12 months for 5 years. Colonoscopy was performed within 1 year of surgery and once every 2–3 years thereafter. Additional radiologic examination or tissue biopsy was performed in patients whom recurrence was suspected during routine follow-up. Locoregional recurrence was defined as any recurrence in the pelvic cavity. Distant metastasis was defined as any recurrence outside the pelvic cavity.

### Statistical analyses

An independent t-test and one-way analysis of variance were used for continuous variables. The chi-square test and Fisher’s exact test were used for categorical variables. The maximally selected log-rank test in R version 3.1.2 (R Development Core Team, Vienna, Austria) was used to investigate proper cut-off values for the NLR [26]. DFS duration was defined as the time from the start of preoperative CRT to recurrence, death, or the last follow-up date. OS duration was defined as the time from the start of CRT to death or last follow-up date. Locoregional recurrence-free survival (LRFS) and distant metastasis-free survival (DMFS) were defined as the time from the start of preoperative CRT to the occurrence of locoregional recurrence or distant metastasis, respectively. The Kaplan-Meier method was used to analyze DFS, OS, LRFS and DMFS. Univariate and multivariate analyses were performed using a Cox proportional hazards model. All variables which were significantly associated with DFS in the univariate analysis were included in the multivariate analysis. Multivariable models were derived using forward stepwise selection. All tests were two-sided and p-values < 0.05 were considered statistically significant.

## Results

### NLR grouping and comparison between NLR groups

A maximally selected log-rank test was performed to identify optimal cut-off values for the NLR. The independent variables were the pre-CRT NLR and post-CRT NLR, and the dependent variable was DFS. The proper cut-off values were identified as 1.75 for the pre-CRT NLR and 5.14 for the post-CRT NLR.

According to the cut-off values of the pre-CRT NLR and the post-CRT NLR, 110 patients were divided into 3 NLR groups: group A (n = 29), a pre-CRT NLR ≤ 1.75 and a post-CRT NLR ≤ 5.14; group B (n = 61), a pre-CRT NLR > 1.75 and a post-CRT NLR ≤ 5.14 (n = 60), or a pre-CRT NLR ≤ 1.75 and a post-CRT NLR > 5.14 (n = 1); and group C (n = 20), a pre-CRT NLR > 1.75 and a post-CRT NLR > 5.14. The median pre-CRT NLR levels in group A, B, and C were 1.22, 2.24, and 2.86, respectively, and the median post-CRT NLR levels in 3 groups were 2.59, 3.13, and 5.86, respectively.

Table 2 shows patients’ characteristics according to their NLR group. Only the pre-CRT NLR and the post-CRT NLR were statistically significant between the 3 NLR groups (both p < 0.001). The levels of pre-CRT NLR and post-CRT NLR were lower in group A than in groups B or C. The other factors such as age, sex, tumor distance from the anal verge, pre-CRT CEA, clinical T stage, clinical N stage, histology, pathologic T stage, pathologic N stage, margin status, LVI, and PNI did not show any significant difference among the 3 NLR groups.

**Table 2:** Patients characteristics according to NLR group.

Characteristics	Group A (n = 29)	Group B (n = 61)	Group C (n = 20)	p value
Age*	59 (40–74)	60 (35–84)	52.5 (27–78)	0.130***
Sex				0.450
Men	17 (58.6%)	42 (68.9%)	15 (75.0%)	
Women	12 (41.4%)	19 (31.1%)	5 (25.0%)	
Pre-CRT NLR *	1.22 (0.53–1.75)	2.24 (1.22–10.63)	2.86 (1.93–4.82)	<0.001***
Post-CRT NLR *	2.59 (0.48–5.07)	3.13 (0.96–21.64)	5.86 (5.15–13.22)	<0.001***
Tumor distance from anal verge				0.678
≤ 5 cm	13 (44.8%)	33 (54.1%)	11 (55.0%)	
> 5 cm	16 (55.2%)	28 (45.9%)	9 (45.0%)	
Pre-CRT CEA, ng/mL				0.256
≤ 5	12 (41.4%)	28 (45.9%)	5 (25.0%)	
> 5	17 (58.6%)	33 (54.1%)	15 (75.0%)	
Clinical T stage				0.344
cT0-2	3 (10.3%)	4 (6.6%)	0 (0.0%)	
cT3-4	26 (89.7%)	57 (93.4%)	20 (100%)	
Clinical N stage				0.344
cN0	3 (10.3%)	4 (6.6%)	0 (0.0%)	
cN1	26 (89.7%)	57 (93.4%)	20 (100.0%)	
Histology				0.319
WD, MD	26 (89.7%)	58 (95.1%)	17 (85.0%)	
PD	3 (10.3%)	3 (4.9%)	3 (15.0%)	
Downstage				0.166
Yes	9 (31.0%)	23 (37.7%)	3 (15.0%)	
No	20 (69.0%)	38 (62.3%)	17 (85.0%)	
pCR				0.467
Yes	2 (6.9%)	8 (13.1%)	1 (5.0%)	
No	27 (93.1%)	53 (86.9%)	19 (95.0%)	
Pathologic T stage				0.546
ypT0-2	9 (31.0%)	23 (37.7%)	5 (25.0%)	
ypT3-4	20 (69.0%)	38 (62.3%)	15 (75.0%)	
Pathologic N stage				0.281
ypN0	22 (75.9%)	43 (70.5%)	11 (55.0%)	
ypN1-2	7 (24.1%)	18 (29.5%)	9 (45.0%)	
Margin status				0.367
Negative	28 (96.6%)	55 (90.2%)	17 (85.0%)	
Positive	1 (3.4%)	5 (9.8%)	3 (15.0%)	
LVI**				0.140
Negative	24 (82.8%)	46 (79.3%)	12 (60.0%)	
Positive	5 (17.2%)	12 (20.7%)	8 (40.0%)	
PNI**				0.419
Negative	24 (85.7%)	45 (78.9%)	14 (70.0%)	
Positive	4 (14.3%)	12 (21.1%)	6 (30.0%)	

*Median, range

**LVI and PNI were evaluated on 107 and 105 patients, respectively, because of missing values

***Independent sample T test. Others: Chi-squared test

Abbreviations: APR = abdominoperitoneal resection, CEA = carcinoembryonic antigen, CRT = chemoradiotherapy, LVI = lymphovascular invasion, MD = moderately differentiated, NLR = neutrophil-lymphocyte count, pCR = pathologic complete response, PD = poorly differentiated, PNI = perineural invasion, post-CRT NLR = NLR values after preoperative CRT, pre-CRT NLR = NLR values before preoperative CRT, SSR = sphincter-saving resection, WD = well differentiated

### DFS, LRFS, DMFS, OS, and failure patterns according to NLR group

**Fig 1:**
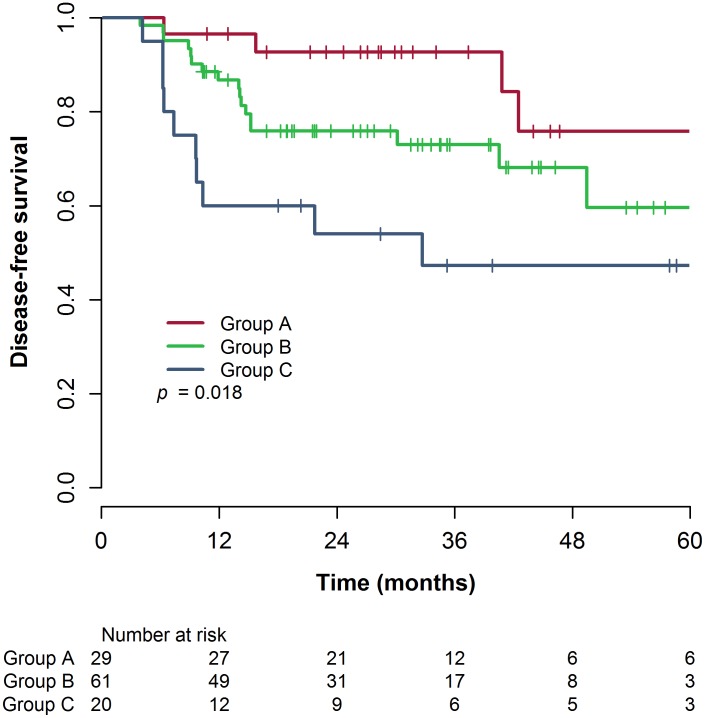
Disease-free survival curves based on the NLR group. Abbreviations: CRT = chemoradiotherapy, NLR = neutrophil-lymphocyte ratio, pre-CRT NLR = NLR values before preoperative CRT, post-CRT NLR = NLR values after preoperative CRT.

The median follow-up duration was 31.1 months. The 3-year DFS rate was 73.1% for all patients. The 3-year DFS rates for groups A, B, and C were 92.7%, 73.0%, and 47.3%, respectively, and there was a statistically significant difference between the NLR groups (p = 0.018) (Fig 1). The 3-year LRFS was 82.5% for all patients. Group A showed a significantly improved LRFS rate compared to groups B and C (96.2% vs. 80.7% vs. 66.5%, for groups A, B, and C, respectively, p = 0.017). The 3-year DMFS was 75.7%. The 3-year DMFS rate was significantly lower in group C than in groups A and B (92.7% vs. 76.4% vs. 51.7%, for groups A, B, and C, respectively, p = 0.040). The 3-year OS rate was 89.9% for all patients. The 3-year OS rates for groups A, B, and C were 96.0%, 85.5%, and 59.8%, respectively, and there was a statistically significant difference between the NLR groups (p = 0.034).

Of the 110 patients, locoregional recurrences were observed in 8 patients (7.3%) and distant metastases occurred in 23 patients (20.9%). The common sites of distant metastases were the liver (10.9%) and lung (9.1%). In group A, distant metastases occurred in 4 patients (13.7%) (liver in 1 patient, lung in 2 patients, and peritoneal seeding, bone, and supraclavicular lymph node in 1 patient). Eleven patients (18.0%) in group B showed distant metastases (liver in 4 patients, lung in 5 patients, para-aortic node in 3 patients, bone in 2 patients, and inguinal node in 1 patient). In group C, 8 patients (40.0%) experienced distant metastases (liver in 7 patients, lung in 3 patients, para-aortic node in 1 patient, inguinal node in 2 patients, peritoneal seeding in 1 patient).

### Clinicopathologic factors that influence DFS

In univariate analysis, DFS was significantly associated with the pre-CRT NLR, post-CRT NLR, pre-CRT CEA, pathologic N stage, PNI, and NLR group (p = 0.040, 0.029, 0.021, 0.038, and 0.027, respectively). In univariate analysis, age and margin status showed a non-significant trend (p = 0.096 and 0.065, respectively). The results of univariate analyses are described in Table 3. Multivariate analysis revealed that NLR group and pre-CRT CEA were significant prognostic factors for DFS (p = 0.028 and 0.035, respectively).

**Table 3:** Clinicopathologic factors that influence DFS.

Variables	No.	3yr-DFS Rate	Univariate analysis		Multivariate analysis	
			Hazard Ratio (95% CI)	p value	Hazard Ratio (95% CI)	p value
Age				0.096		
≤ 60	64	78.5%	1			
> 60	46	66.0%	1.832 (0.899–3.734)			
Sex				0.416		
Men	74	71.9%	1			
Women	36	75.5%	0.716 (0.320–1.601)			
Pre-CRT NLR				0.040		
≤ 1.75	30	92.9%	1			
> 1.75	80	65.7%	3.009 (1.052–8.608)			
Post-CRT NLR				0.029		
≤ 5.14	89	79.1%	1			
> 5.14	21	50.0%	2.324 (1.093–4.945)			
Tumor distance from anal verge				0.529		
≤ 5 cm	57	68.8%	1			
> 5 cm	53	78.1%	0.794 (0.388–1.625)			
Pre-CRT CEA, ng/mL				0.021		0.035
≤ 3	66	77.5%	1		1	
> 3	44	68.0%	2.703 (1.163–6.280)		2.478 (1.064–5.772)	
Histology				0.365		
WD, MD	101	74.8%	1			
PD	9	55.6%	1.626 (0.568–4.652)			
Downstage				0.127		
Yes	35	84.2%	1			
No	75	67.7%	2.004 (0.820–4.896)			
pCR				0.503		
Yes	11	81.8%	1			
No	99	72.2%	1.632 (0.389–6.849)			
Pathologic T stage				0.188		
ypT0-2	37	82.6%	1			
ypT3-4	73	68.1%	1.762 (0.758–4.095)			
Pathologic N stage				0.038		
ypN0	75	79.6%	1			
ypN1-2	35	59.6%	1.972 (0.971–4.003)			
Margin status				0.065		
Negative	100	75.7%	1			
Positive	10	45.0%	2.490 (0.946–6.553)			
LVI*				0.287		
Negative	82	75.6%	1			
Positive	25	62.6%	1.525 (0.701–3.319)			
PNI*				0.046		
Negative	83	76.5%	1			
Positive	22	57.3%	2.114 (1.012–4.414)			
NLR group				0.027		0.028
Group A	29	92.7%	1		1	
Group B	61	73.0%	2.338 (0.786–6.958)		2.503 (0.841–7.448)	0.099
Group C	20	47.3%	4.658 (1.459–14.874)		4.350 (1.361–13.901)	0.013

*LVI and PNI were evaluated on 107 and 105 patients, respectively, because of missing values

Abbreviations: APR = abdominoperitoneal resection, CEA = carcinoembryonic antigen, CI = confidence interval, CRT = chemoradiotherapy, DFS = disease free survival, LVI = lymphovascular invasion, MD = moderately differentiated, NLR = neutrophil-lymphocyte ratio, pCR = pathologic complete response, PD = poorly differentiated, PNI = perineural invasion, post-CRT NLR = NLR values after preoperative CRT, pre-CRT NLR = NLR values before preoperative CRT, SSR = sphincter-saving resection, WD = well differentiated

## Discussion

Inflammation has been suggested as a hallmark of cancer [13,27]. A systemic inflammatory response leads to angiogenesis, inhibition of apoptosis, and DNA damage. The NLR is a factor related to systemic inflammation [28,29]. A high NLR means a relatively elevated neutrophil count and depletion of lymphocytes. Elevated neutrophils secrete serum vascular endothelial growth factor and various proteases [30]. This tumor-promoting microenvironment facilitates tumor invasion and metastasis. Moreover, lymphocytes play a role in adaptive immunity against cancer cells. Depletion of lymphocytes means weakened anti-tumor immunity. Many studies reported that a high NLR is associated with a poor survival outcome in multiple solid tumors [8–11]. In HCC patients, the pre-treatment NLR was a significant predictor of recurrence and survival in various treatment settings, including surgical resection and trans-arterial chemoembolization [14,15,31,32]. The prognosis of gastric cancer, renal cancer, pancreatic cancer, and intrahepatic cholangiocarcinoma were also associated with the NLR [8–11].

Carruthers et al. were the first to suggest that that pre-CRT NLR is a predictive factor for prognosis in patients with rectal cancer who received preoperative CRT [18]. Patients with a pre-CRT NLR ≥ 5 showed a significantly worse OS and DFS. The authors stated that the effect of the NLR on prognosis was independent of margin status and downstaging. Subsequent studies have showed the usefulness of the pre-CRT NLR as a prognostic factor in patients with rectal cancer who received preoperative CRT. Nagasaki et al. analyzed 201 patients with locally advanced low rectal cancer [19]. The high pre-CRT NLR group included patients who had a pre-CRT NLR ≥ 3.0. A high pre-CRT NLR was a significant adverse prognostic factor for OS. Another study by Shen et al. reported the prognostic significance of the pre-CRT NLR [33]. The 5-year DFS rate in patients with an NLR < 2.8 was 62.5%, which was significantly different compared to 33.4% in patients with a NLR ≥ 2.8 (p = 0.032).

Our study showed the prognostic value of the pre-CRT NLR in locally advanced rectal cancer, which was consistent with previous studies. Patients with a low pre-CRT NLR showed a significantly increased 3-year DFS rate compared to those with a high pre-CRT NLR (92.9% vs. 65.7%, p = 0.040). Furthermore, we suggest that post-CRT NLR also has a prognostic value. In univariate analysis, both the pre-CRT NLR and the post-CRT NLR were significantly associated with DFS. Patients with a high post-CRT NLR had a decreased 3-year DFS rate compared to patients with a low post-CRT NLR (79.1% vs. 50.0%, p = 0.029). NLR group was a factor which combines both the prognostic value of pre-CRT and that of post-CRT NLR. The 3-year DFS rates of the 3 NLR groups showed a sequentially decreasing pattern from group A to group C (92.7% vs. 73.0%, vs. 47.3%, for groups A, B, and C, respectively, p = 0.018). After adjusting for other clinicopathological factors, NLR group was identified as an independent predictor of prognosis. To the best of our knowledge, this is the first study to show the prognostic impact of the post-CRT NLR in patients with rectal cancer who received preoperative CRT. In addition, this study is the first to demonstrate the prognostic significance of using a combination of both the pre- and post-CRT NLR in these patients.

To predict prognosis according to the NLR, the statistical method for determination of the cut-off value is important. A normal upper limit of the NLR to predict prognosis has not been clearly defined in rectal cancer. Various values were used to define the high and low NLR groups in previously published studies. The previously mentioned report by Carruthers et al. defined a high pre-CRT NLR of ≥ 5.0, adopting the cut-off value of previous studies [18,34]. However, the referenced studies investigated other primary sites or metastatic colorectal cancer. Two subsequent studies performed receiver operating characteristic (ROC) curve analyses. Nagasaki et al. selected a pre-CRT NLR of 3.0, which was a historical cut-off value, nearest to the calculated figure of 3.2 based on ROC curve analysis. [19]. Shen et al. also determined the cut-off value of the NLR using ROC curve analysis; they defined a pre-CRT NLR ≥ 2.8 as the high NLR group [33]. T outcome variable in the ROC curve analyses of both studies was death. The analysis could show the optimal point to discriminate surviving patients from the deceased patients, but could not discriminate between early death and late death. In this study, to determine the optimal cut-off value for the NLR, a maximally selected log-rank test was used. In this statistical analysis, the survival duration also was also used as a variable. In this study, we identified the cut-off values for NLR (pre-CRT NLR of 1.75 and post-CRT NLR of 5.14), and 3 NLR groups were defined and evaluated. We suggest that our cut-off values are optimal for predicting prognosis because of the valid statistical method and the large differences in DFS between the NLR groups.

In this study, significance differences in DMFS were shown between the groups. The 3-year DMFS rate was significantly lower in group C than in groups A or B (p = 0.040); the differences were 41.0% and 24.7%, respectively. The absolute rates of distant metastases were also lower in group C than in groups A or B, and the differences were 16.3% and 12.0%, respectively. For these reasons, the NLR could be an indicator of occult metastasis and consequential systemic failure. Therefore, especially in group C, careful evaluation of systemic failure is needed, and more aggressive adjuvant chemotherapy should be considered.

These results should be cautiously interpreted due to the relatively small number of cases and the retrospective nature of this study. In addition, the NLR could be influenced by infection, drugs, or non-tumorous diseases such as, rheumatoid disease, coronary artery disease, or metabolic syndrome [22–24]. We excluded patients with these conditions from our study cohort. However, it is still possible that patients with other conditions that could change the NLR were not completely excluded. Although our cut-off values were obtained using valid statistical analyses, they are not sufficient to be generally accepted, and therefore, they need to be validated in further large-scale studies.

In conclusion, we describe the prognostic value of both the pre-CRT NLR and the post-CRT NLR in patients treated with preoperative CRT and curative TME followed by adjuvant chemotherapy. A persistently elevated post-CRT NLR may be an indicator of an increased risk of distant metastasis. Further studies are needed to confirm the prognostic value of the pre-CRT NLR and the post-CRT NLR, together with their proper cut-off values.
